# Downscaling of Organic Field‐Effect Transistors based on High‐Mobility Semiconducting Blends for High‐Frequency Operation

**DOI:** 10.1002/smtd.202400546

**Published:** 2024-08-06

**Authors:** Tommaso Losi, Fabrizio Antonio Viola, Elda Sala, Martin Heeney, Qiao He, Hans Kleemann, Mario Caironi

**Affiliations:** ^1^ Center for Nano Science and Technology Istituto Italiano di Tecnologia Via Rubattino 81 Milano 20134 Italy; ^2^ Department of Electrical and Electronic Engineering University of Cagliari via Marengo Cagliari 09123 Italy; ^3^ Department of Energy Politecnico di Milano, Via Lambruschini 4a Milan 20156 Italy; ^4^ Department of Chemistry and Centre for Processable Electronics Imperial College London London W12 0BZ UK; ^5^ Dresden Integrated Center for Applied Physics and Photonic Materials (IAPP) Technische Universität Dresden Nöthnitzer Straße 61 01062 Dresden Germany

**Keywords:** doping, high‐frequency transistors, organic electronics, organic semiconductors, organic transistors

## Abstract

Small molecule/polymer semiconductor blends are promising solutions for the development of high‐performing organic electronics. They are able to combine ease in solution processability, thanks to the tunable rheological properties of polymeric inks, with outstanding charge transport properties thanks to high crystalline phases of small molecules. However, because of charge injection issues, so far such good performances are only demonstrated in ad‐hoc device architectures, not suited for high‐frequency applications, where transistor dimensions require downscaling. Here, the successful integration of the most performing blend reported to date, based on 2,7‐dioctyl[1] benzothieno[3,2‐*b*][1]benzothiophene (C_8_‐BTBT) and poly(indacenodithiophene‐*co*‐benzothiadiazole) (C_16_IDT‐BT), in OFETs characterized by channel and overlap lengths equal to 1.3 and 1.9 µm, respectively, is demonstrated, enabling a transition frequency of 23 MHz at ‐8 V. Two key aspects allowed such result: molecular doping, leading to width‐normalized contact resistance of only 260 Ωcm, allowing to retain an apparent field‐effect mobility as high as 3 cm^2^/(Vs) in short channel devices, and the implementation of a high capacitance dielectric stack, enabling the reduction of operating voltages below 10 V and the overcoming of self‐heating issues. These results represent a fundamental step for the future development of low‐cost and high‐speed printed electronics for IoT applications.

## Graphical abstract

This work reports the successful integration of a high‐mobility organic semiconducting blend in downscaled transistors reaching a transition frequency of 23 MHz at ‐8 V. The devices show also good air and shelf‐life stability. These results demonstrate the great potential of organic blends for the development of high‐speed solution‐processed electronics.

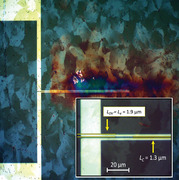

## 1 Introduction

Cost‐effective electronics and sensors integration in everyday life smart objects is foreseen as a key‐aspect for a life‐quality improvement in the upcoming future. Distributed electronics implemented in our normal routine will smoothly change the relation that we have with technology. In particular, the creation of an electronic environment formed by wireless communicating devices known as the Internet of Things (IoT) will be helpful in many fields such as healthcare, food and pharmaceutical industry, agriculture, security, entertainment and the energy sector, as well as space applications.^[^ ^1^, ^2^, ^3^, ^4^, ^5^, ^6^ ^]^ In this scenario, organic electronics offers the advantage to be compatible with scalable large‐area, low‐cost and high‐throughput solution‐based additive fabrication processes, along with the possibility of using flexible and conformable substrates, highly desirable for wearable and light‐weight devices. For this reason, a lot of effort has been dedicated to improve electrical performances of organic field‐effect transistors (OFETs), especially in terms of charge carrier mobility, which has approached that of solution‐processed low temperature metal oxides (≈10 cm^2^/(Vs)).^[^ ^7^, ^8^, ^9^, ^10^ ^]^

In particular, the use of organic blends based on small molecules and polymers has proved to be an effective strategy to ease the solution processability over large‐areas of small molecules, which typically display a higher mobility than that of polymers, but also a narrower processing window. Thanks to the high molecular weight of polymers, organic blends can be tuned over wide rheological ranges, making them compatible with a broad set of deposition techniques, from large‐area blade coating to inkjet printing.^[^ ^11^, ^12^, ^13^, ^14^, ^15^ ^]^ At the same time, phase segregation between the two materials can be exploited to form highly crystalline small molecule phases, allowing to largely retain high mobilities.^[^ ^16^, ^17^ ^]^ Such good electronic properties promise in principle the realization of printed organic electronics operating at high frequencies, as required for efficient wireless communication purposes. However, so far, charge transport of the best organic blends have been probed only in ad‐hoc transistor architectures, typically characterized by long channels, high operating voltages and high capacitive parasitism, therefore completely precluding their use for high‐frequency applications. To achieve operational speeds compatible with wireless communication, transistors critical dimensions, such as channel and overlap length, require downscaling. In fact, the transition frequency (*f_T_*), the most widely adopted figure of merit used to characterize the dynamic performance of a transistor, is related to its geometrical and electrical parameters according to the following expression:^[^ ^18^ ^]^

(1) $$f_{T}=\frac{g_{m}}{2\pi C_{g}}=\frac{\mu_{eff}\left(V_{gs}-V_{T}\right)}{2\pi L_{C}\left(\frac{2}{3}L_{C}+2L_{OV}\right)}$$

where *g_m_* is the transistor transconductance, *C_g_* the total gate capacitance, *µ_eff_* the effective charge carrier mobility,^[^ ^19^ ^]^ *V_T_* the threshold voltage, *L_C_* and *L_OV_* the channel and overlap lengths, respectively. Unfortunately, severe performance degradation occurs in downscaled OFETs, where charge injection properties from contact metals to semiconductor start to become dominant over transport in the channel owing to contact resistance issues.^[^ ^20^ ^]^ Several factors contribute to such high *R_C_W* values. In staggered transistors, the highly resistive bulk portion of the active material, through which carriers must travel across to reach the modulated channel, contributes to the so called access resistance (*R_C bulk_*).^[^ ^21^, ^22^, ^23^ ^]^ A typical mitigation strategy for the latter contribution comes from the reduction of the semiconductor thickness. However, the energetic disorder and the mismatch between the energy levels at the metal semiconductor interface introduce a critical contribution to contact resistance over different transistors architectures (*R_C int_*). A typical strategy used to improve the injection efficiency in this case is to modify the electrode surface with charge injection layers.^[^ ^24^, ^25^, ^26^ ^]^ An example is the use of self‐assembled monolayers (SAMs), able to tune the work function of metals, such as gold and silver, in order to match the energy levels of the selected semiconductor.^[^ ^26^, ^27^, ^28^, ^29^ ^]^ Another powerful tool in the effort to further reduce contact effects comes from molecular doping.^[^ ^30^, ^31^, ^32^, ^33^, ^34^ ^]^ Yet, several adverse effects, such as dopant segregation and increase of energetic disorder, typically limit doping level in OFETs to ≤ 1 mol%, reducing the benefit of such strategy.^[^ ^30^, ^35^, ^36^, ^37^ ^]^

In this work we demonstrate high‐frequency operation of low‐voltage, downscaled FETs based on a solution‐processable, high‐performing organic semiconducting blend. The adopted blend is based on the small molecule C_8_‐BTBT (2,7‐dioctyl^[^ ^1^ ^]^ benzothieno[3,2‐*b*][1]benzothiophene) and the polymer C_16_IDT‐BT (poly(indacenodithiophene‐*co*‐benzothiadiazole)), leading to one of the best performing poly‐crystalline organic semiconductors known to date.^[^ ^38^ ^]^ High‐frequency operation was achieved thanks to careful engineering of injection and transport properties when downscaling critical OFET dimensions. In particular, we show that it is possible to molecularly dope the blend at unusual high doping levels using the fluorinated fullerene derivative C_60_F_48_ without degrading transport properties, leading to an improvement of both mobility and injection performances up to 5 mol%. With this approach it was possible to reduce width‐normalized contact resistance down to 260 Ωcm, achieving the lowest reported value for a OFET based on a polymer semiconductor. Thanks to the injection improvement, aggressive downscaling of the channel length and of the overlap length, down to 1.3 and 1.9 µm, respectively, was possible, preserving an apparent field‐effect mobility (*µ*_app_) of 3 cm^2^/(Vs). Furthermore, the use of a very thin organic dielectric stack (≈80 nm) was of crucial importance to lower operating voltages below 10 V, alleviating thermal degradations incurring during the device operation. This strategy allowed to achieve a transition frequency of 23 MHz at *V_gs_* = *V_ds_* = ‐8 V, corresponding to a voltage‐normalized transition frequency (*f_T_*/*V*) of 2.8 MHz/V. The optimized OFETs proved also promising air and shelf‐life stability (up to 6 weeks) even without any encapsulation.

Our results demonstrate the viability of using high‐performance and solution‐processable organic blend semiconductors in downscaled devices. These findings are of utmost relevance for future developments of low voltage and high speed organic electronic building‐blocks serving the application of large‐area printed electronics in wireless sensing and IoT applications.

## 2 Results and Discussion

**Figure 1:**
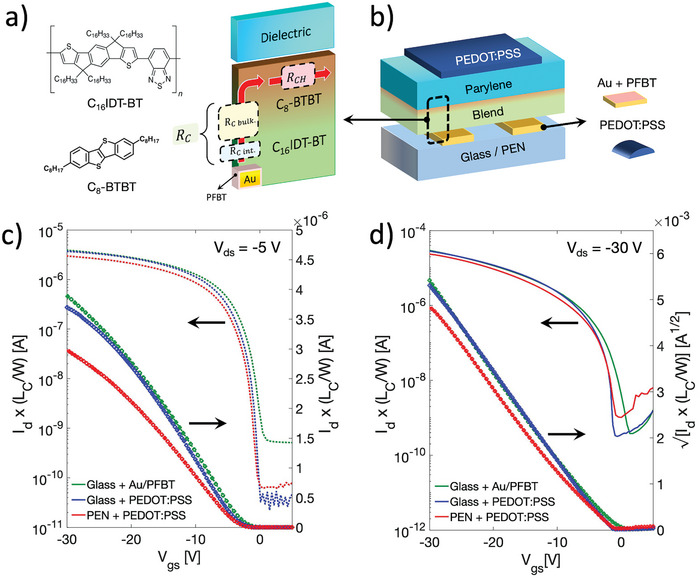
a) Chemical structures of C_16_IDT‐BT and C_8_‐BTBT and scheme of their phase segregation in the solid‐state, highlighting the different roles of the two semiconductors when integrated in top‐gate bottom‐contacts OFETs. b) Sketch of the device architecture. c) Linear and d) saturation width‐normalized transfer characteristic curves of OFETs based on C_8_‐BTBT:C_16_IDT‐BT blend with different contact and substrate types.

Previous studies demonstrated that blending the high‐performing small molecule C_8_‐BTBT with the donor‐acceptor copolymer C_16_IDT‐BT resulted in an enhancement of field‐effect mobility in top‐gate bottom‐contacts (TGBC) transistors if compared to that of the two neat materials in the form of polycrystalline and amorphous‐like thin films, respectively.^[^ ^39^ ^]^ The excellent electrical performance of the blend is related to the vertical phase segregation occurring between the two semiconductors, which leads to the formation of a thin and highly crystalline phase at the top interface with the dielectric, rich in small molecule, mainly responsible for the channel conduction, while the polymer is mostly segregated at the bottom, with a predominant role in charge injection (**Figure** **1**a).^[^ ^40^ ^]^ Recently, it was demonstrated that the polymer plays an active role also in charge transport, improving the interconnectivity among crystalline domains of the small molecules.^[^ ^40^ ^]^ However, up to now the electrical properties of the blend were only probed in ad‐hoc, high‐voltage OFETs architectures, not ideal for high‐frequency operation.

As a first step in order to favor a correct downscaling of the transistors and the reduction of the operation voltages, we tackled the replacement of low‐capacitance dielectric layers, adopted so far with the high‐performance blend, with high‐capacitance ones in relatively long channel (*L_C_* = 80 µm) TGBC OFETs (Figure 1b). Since the commonly adopted Cytop and Teflon^[^ ^34^, ^38^, ^39^, ^40^, ^41^, ^42^ ^]^ do not allow the implementation of very thin layers because of high leakage currents and relatively low breakdown voltages, we tested Parylene‐C as alternative insulator, as it offers higher robustness and lower leakage currents. We first verified the compatibility of the insulator with the C_8_‐BTBT:C_16_IDT‐BT pristine, that is, non‐doped, blend starting from a relatively thick film of 250 nm, which is commensurate with the relaxed channel length adopted in such optimization experiments. The blend solution was formulated according to previous optimizations at a concentration of 10 g L^−1^ of C_8_‐BTBT:C_16_IDT‐BT in a 1:4 wt.% ratio,^[^ ^39^, ^42^ ^]^ and was deposited by spin‐coating to form 70 nm thick films (measured by mechanical profilometer). To provide an ample spectrum of the potential of the blend for different OFETs configurations, we initially explored the use of both glass and flexible poly(ethylene 2,6‐naphthalate) (PEN) substrates, as well as the use of electrodes made of evaporated gold, functionalized with a self‐assembled monolayer of 2,3,4,5,6‐pentafluorothiophenol (PFBT) to improve the hole injection, and inkjet‐printed electrodes made of PEDOT:PSS. Such tests were performed in order to assess the possibility of obtaining well‐performing devices even when using substrates and electrodes with different properties, for example in terms of surface roughness. In Figure 1c,d, a comparison between representative width‐normalized transfer characteristic curves of long channel OFETs is shown, for both linear (*V_ds_* = ‐5 V) and saturation regime (*V_ds_* = ‐30 V), respectively. The data regarding the gate currents are presented in Figure S1 (Supporting Information), while in Figure S2 (Supporting Information) the corresponding output curves are reported. The transfer curves present in Figure 1 are characterized by a good p‐type behavior with on/off ratio ≥10^4^ and threshold voltages close to zero. Values of maximum apparent saturation field‐effect mobility (Figure S3a, Supporting Information) as high as 7.5 and 6 cm^2^/(Vs) with reliability factors (*r_sat_*)^[^ ^19^, ^43^ ^]^ equal to ≈95% are derived for devices based on glass and PEN substrates, respectively. Whereas, the maximum apparent linear mobilities extracted are equal to: 3.5 cm^2^/(Vs) (glass substrate, Au/PFBT electrodes), 3 cm^2^/(Vs) (glass, PEDOT:PSS), and 2.5 cm^2^/(Vs) (PEN, PEDOT:PSS), respectively (Figure S3b, Supporting Information), with a corresponding reliability factor of ≈70%. The lower reliability factor with respect to the saturation regime, and the non‐negligible deviation from the ideal linear behavior of the drain current at high gate voltage, are both indications of contact effects.^[^ ^19^ ^]^ Using the Y‐function method^[^ ^47^ ^]^ (Figure S4, Supporting Information), width‐normalized contact resistances equal to 7.8, 8.3 and 9 kΩcm are estimated, respectively. Such values are in agreement with what previously reported for C_8_‐BTBT:C_16_IDT‐BT pristine blend in similar transistor configurations.^[^ ^34^ ^]^ Yet, they introduce non‐negligible effects in the device transfer characteristic due to the high mobility of the semiconductor.

**Figure 2:**
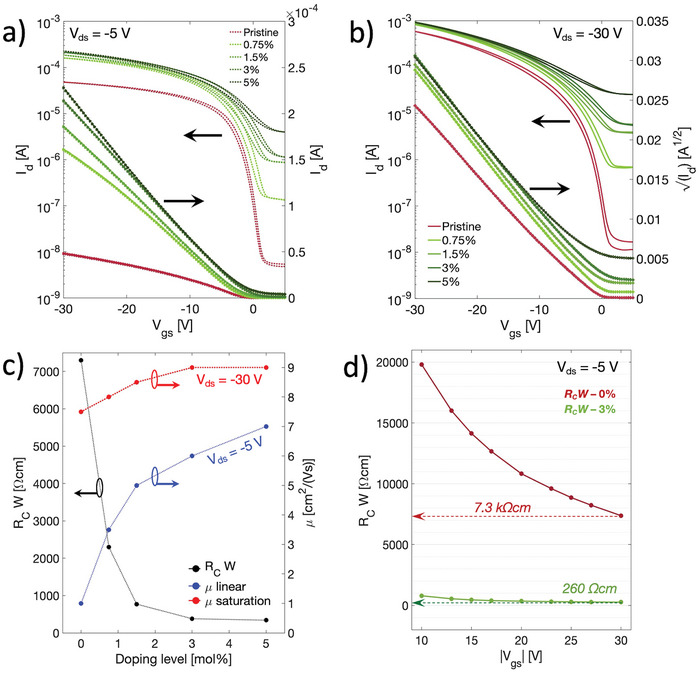
Transfer characteristic curves in linear a) and saturation b) regimes of long channel transistors (*L_C_* = 80 µm) having different doping levels of C_60_F_48_. Channel width is 2 mm. c) Trend of saturation and linear apparent mobility in long channel devices (*L_C_* = 80 µm) and of width‐normalized contact resistance width doping. d) Width‐normalized contact resistances of the pristine and doped blend‐based OFETs extracted with TLM.

After validating the compatibility of Parylene‐C as a dielectric for high‐mobility blend‐based OFETs, we moved to the improvement of charge‐injection efficiency, a necessary step to avoid severe short‐channel effects. To this aim, among the explored configurations, we selected the one characterized by a lower starting *R_C_W*, that is, glass substrate and Au/PFBT electrodes. To reduce contact resistance, we introduced the fluorinated fullerene C_60_F_48_ as molecular dopant in the blend, which was already demonstrated to be effective in increasing mobility and reducing contact effects.^[^ ^34^ ^]^ In **Figure** **2**a,b transfer curves in linear and saturation regime of long channel (*L_C_* = 80 µm) OFETs having different doping levels (from 0.75 to 5 mol%) are shown. The corresponding mobility curves are presented in Figure S5a,b (Supporting Information), while the output characteristics are reported in Figure S6 (Supporting Information). Figure 2c shows the trend of the saturation and linear apparent field‐effect mobility and that of width‐normalized contact resistance with doping concentration. Contact resistance was extracted using the Transfer Length Method (TLM) (Figure S7, Supporting Information). Increasing the doping level, a noticeable degradation of the on/off current ratios, owing to the higher transistor off currents, takes place. The latter is a direct effect of the increase in bulk, non‐gateable, electrical conductivity of the thin film. It would be detrimental for logic applications, while it is more acceptable for analog applications, where the device would mostly work in saturation regime operating with small signal input.^[^ ^48^, ^49^ ^]^ Beyond 5 mol%, a too weak current modulation establishes, setting a higher limit for the dopant concentration in transistors. Despite such effects, it is possible to notice a general improvement in the transistors on currents with increased doping level, leading to a consequential increase in both saturation and liner apparent mobilities. In particular, mean value of *µ*_app_ goes from 7.5 to 9 cm^2^/(Vs) in saturation regime, and from 2.5 to 7 cm^2^/(Vs) in linear regime, respectively. The higher gain in linear mobility with doping is directly related to the reduction of contact resistance, since at lower applied lateral voltages devices typically suffer more injection limitations. The width‐normalized contact resistance is reduced from 7.3 kΩcm in the case of the pristine blend down to 260 Ωcm when doped with ≥3 mol% of C_60_F_48_. Such a result can be ascribed to an increased charge carrier density, which causes a shrinking of the depletion region width and a lowering of the height of the injection barrier by shifting the Fermi level of the semiconducting layer, reducing the interfacial contribution of contact resistance (*R_C int_*).^[^ ^36^, ^40^, ^50^ ^]^ In addition, excess charges introduced by doping are also able to fill trap states at the contacts‐semiconductor interfaces and in the bulk polymer‐rich layer, reducing both *R_C int_* and the access contribution of contact resistance (*R_C bulk_*).^[^ ^36^, ^40^ ^]^ Such an effect is often correlated to a reduction of the *V_gs_* dependence of *R_C_* because the charge transport through the trap‐rich regions is highly affected by the semiconductor charge carrier density and gate voltage.^[^ ^33^, ^36^, ^51^ ^]^ Figure 2d shows the comparison between *R_C_W* of pristine and doped blend‐based transistors. It is possible to appreciate that contact resistance has a reduced *V_gs_* dependence upon doping, suggesting a trap‐filling effect as one possible contribution of the injection improvements. Besides, also the *V_gs_* independent contribution of contact resistance seems to be reduced. Such component of *R_C_W* is the main responsible for the deviation from the linear behaviour of *I_ds_* in linear regime.^[^ ^51^ ^]^ As shown in Figure 2a, drain currents have indeed an improved linearity in the transfer characteristics with doping, which is also reflected in a higher reliability factor of ≈90%.

It is worth noticing that in the whole doping range explored, the transistor performances are continuously enhanced with doping. This is different from what is typically observed in literature, where for doping concentrations above or close ≈1 mol% detrimental effects on charge transport are often reported because of increased structural disorder in the film.^[^ ^30^, ^35^, ^36^, ^37^ ^]^ One could speculate that, at least up to 5 mol%, the excess free charges can compensate for any eventual trap states caused by the increased disorder. Yet, the three‐component blend offers more specific mechanisms that may take place. It is known that, because of its steric hindrance, the dopant tends to be segregated into the more amorphous‐like polymer‐rich phase, rather than in the highly ordered phase of small molecule, as already reported in ref. [39] Therefore, since the polymer dominates charge injection, the dopant is expected to have a higher impact on the OFETs contact resistance rather than on the channel conductivity, dominated by the small molecule. Moreover, while UV–vis spectroscopy confirms an unambiguous interaction between the polymer and the dopant, with the formation of a polaron band indicating doping of the polymer, no clear sign of an interaction with the small molecule is observed (Figure S8, Supporting Information). While further studies are required to clarify this aspect, we have clearly evidenced that the blend offers a broader concentration window where doping is beneficial for injection and does not interfere with charge transport.

**Figure 3:**
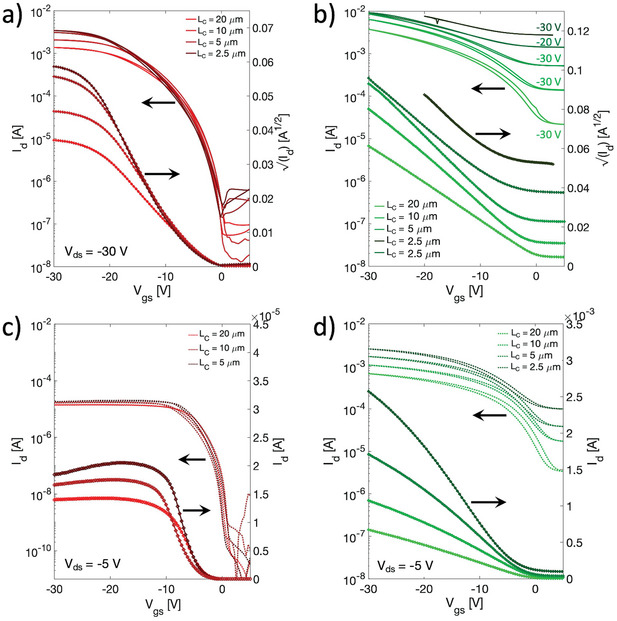
Saturation a) and linear c) transfer characteristics of pristine short channel OFETs. Saturation b) and linear d) transfer curves of 3% mol. doped short channel OFETs. The channel width is 2 mm.

After studying the effect of molecular doping on reference long channel devices, we moved to the exploration of the scaling behavior of OFETs with reduced channel length. In shorter channel OFETs, where the transistor off current is further increased by reducing the device critical dimension, doping levels above 3 mol% could not be considered to allow a minimum on/off ratio. In fact, with 3 mol%, the transistor on/off ratio at a channel length of 10 µm is already below 10^2^ in saturation regime (**Figure** **3**b). The results for transistors with channel lengths of 20, 10, 5, and 2.5 µm, are shown in Figure 3, where a comparison of saturation and linear transfer curves is presented for both pristine and doped devices, respectively. In saturation regime, for the undoped transistors (Figure 3a) a marked suppression of the drain current from the ideal slope is evident at high gate voltages, which leads to a reduction of both mobility and channel transconductance (*g_m_*) (Figure S9a–c, Supporting Information). For example, for *L_C_* = 10 µm, *µ*_app_ reduces from a maximum of 6 cm^2^/(Vs), at low *V_gs_* values, to ≈0.1 cm^2^/(Vs), at high *V_gs_* values, while *g_m_* goes from 0.15 to 0.013 mS at high gate voltages. Such non‐ideality is reflected in a poor reliability factor of 40%. Also, Figure S9c (Supporting Information) clearly shows no gain in transconductance with a channel length reduction as a consequence of contact limitations. With 3 mol% of doping (Figure 3b), both *g_m_* and field‐effect mobility show a reduced *V_gs_* dependence (Figure S9b–d, Supporting Information), with an improved *r_sat_* of 95% for all channel lengths. Yet, *I_ds_* does not scale perfectly with *L_C_* and consequently the apparent mobility is reduced when the transistor channel is shortened, from 7 cm^2^/(Vs) for *L_C_* = 20 µm to 2 cm^2^/(Vs) for *L_C_* = 2.5 µm. However, despite of such limitation, the lower *R_C_W* allows to exploit channel downscaling to increase the current and the device transconductance, which goes from 0.2 to 0.45 mS from *L_C_* = 20 µm to *L_C_* = 2.5 µm, respectively (Figure S9d, Supporting Information). In Figure 3c,d a comparison between the linear transfer curves is presented. For the pristine transistors a similar behavior to that of the saturation regime can be observed. With 3 mol% of doping a reliable linear apparent mobility as high as 2.5 cm^2^/(Vs), with a reliability factor of 85%, is achieved for *L_C_* = 2.5 µm (Figure S10a,b, Supporting Information for comparing undoped and doped case, respectively).

**Figure 4:**
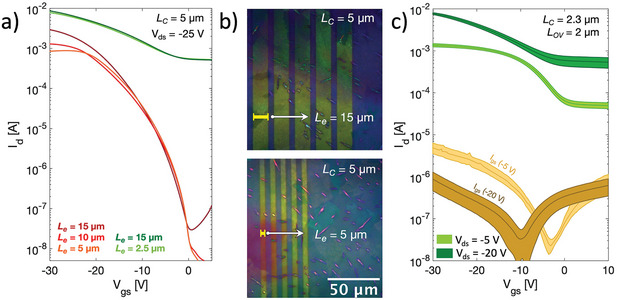
a) Comparison of saturation transfer curves of OFETs having same channel length but different electrode length, for both pristine (red curves) and doped (green curves) blend. b) Example of optical polarized images of source and drain bottom electrodes of two transistors with different contact length. The two figures have the same scale bar. c) Average transfer curves, with standard deviation, in linear and saturation regime for ten different downscaled doped (3 mol% of C_60_F_48_) OFETs having a channel length of 2.3 µm and an overlap length of 2 µm. Channel width is 2 mm.

Having achieved apparent mobilities in excess of 1 cm^2^/Vs in micron‐scale channels thanks to improved charge injection is a critical result towards high‐frequency operation of blend‐based OFETs. Improved charge injection can also favor the reduction of another important geometrical dimension in high‐frequency transistors, that is the overlap length between the gate electrode and source and drain ones (*L_OV_*, see Equation 1). In fact, the injection efficiency depends on *L_OV_*, and the characteristic transfer length (*L_T_*) determines the effective injection area of the contacts, therefore it sets a lower limit to *L_OV_*.^[^ ^52^ ^]^ We computed *L_T_* using the current crowding model, which was revealed to be a reliable method for staggered geometries.^[^ ^22^, ^25^, ^52^, ^53^ ^]^ We derived values as low as 22 and 1.8 µm for the pristine and doped (3 mol%) blend, respectively (Figure S11, Supporting Information). To confirm the theoretical estimations, we fabricated transistors with fixed channel length (*L_C_* = 5 µm) but different electrode length (*L_e_*), which defines the overlap length of the devices (*L_OV_* = *L_e_*, **Figure** **4**b). As presented in Figure 4a, the pristine OFETs show significant performance degradation when *L_e_* is reduced below 15 µm, since *L_OV_* < *L_T_*, while little differences are observed in doped transistors when *L_e_* is reduced down to few micrometers, since *L_OV_* > *L_T_* still holds. In Figure 4c, we propose a statistical analysis over ten different downscaled doped OFETs characterized by *L_C_* = 2.3 µm and *L_OV_* = *L_e_* = 2 µm, proving a good reproducibility of the device performances. In Figure S12 (Supporting Information) a plot summarizing the variation of the most relevant figure‐of‐merits is presented.

Despite these results, the fabricated downscaled devices present a severe bias instability, and cannot be consistently characterized. Because of the high current density (few A mm^−2^) and the high overdrive voltages, the thermal power density dissipated per unit area is around tens of W mm^−2^, which is expected to considerably raise the temperature of the transistor.^[^ ^54^ ^]^ Figure S13a (Supporting Information) shows considerable thermal degradations in OFETs with *L_C_* and *L_e_* below 5 µm. Figure S13b (Supporting Information), instead, shows how the degradation caused by the device self‐heating is irreversible as the drain current continuously decreases during consecutive measurements, even when the applied voltages are slightly reduced (both *V_gs_* and *V_ds_* < ‐30 V). In the example presented in Figure S13b (Supporting Information), after six measurements *I_ds_* and *g_m_*/*W* decreased by ≈30% and 52%, respectively.

**Figure 5:**
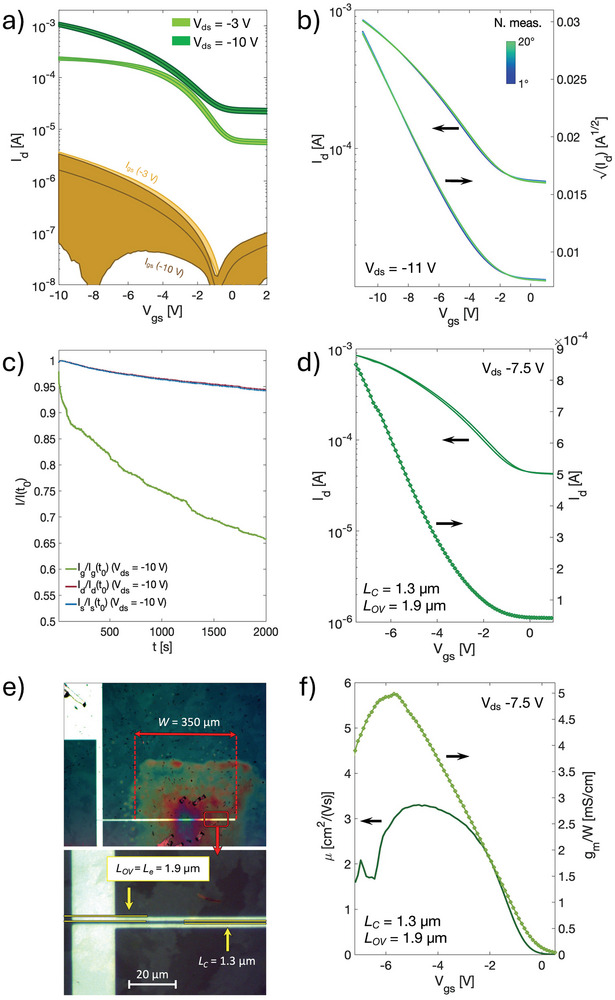
a) Average linear and saturation transfer curves, with standard deviation, of ten different short channel doped (3 mol% of C_60_F_48_) OFETs (*L_C_* = 2.5 µm) with a double stack dielectric made of 25 nm of PVF and 50 nm of Parylene. The channel width is 600 µm. b) Cyclic measurements and c) bias‐stress test performed on a 2.5 µm channel OFET in saturation regime. d) Saturation transfer curve, e) optical polarized image, and f) relative apparent saturation mobility and width‐normalized channel transconductance of a downscaled transistor with channel length of 1.3 µm and overlap length equal to 1.9 µm. Channel width is 350 µm.

To overcome thermal degradation issues, the 250 nm thick Parylene layer was replaced with a thinner dielectric stack, formed by an ultra‐thin pinhole‐free nanosheet made of poly(vinyl formal) (PVF) of ≈25 nm and an additional 50 nm thick layer of Parylene, for a total dielectric thickness of ≈75 nm and a gate dielectric capacitance of ≈35 nF cm^−2^. The PVF film is solution‐processed by delamination in water according to ref.[55] and it shows low leakage current on its own, as shown in Figure S14 (Supporting Information). The additional Parylene layer was used to further reduce leakage currents and increase the success rate of device fabrication. Because of the higher dielectric capacitance compared to the 250 nm thick Parylene film (≈10 nF cm^−2^), operating voltages can be reduced from ‐30 to ‐10 V, therefore reducing of nine times the dissipated power density from the transistor. In **Figure** **5**a, the average linear (*V_ds_* = ‐3 V) and saturation (*V_ds_* = ‐10 V) transfer characteristic curves of ten short channel transistors (*L_C_* = 2.5 µm) with a double stack dielectric layer are shown, from which average linear and saturation apparent mobilities as high as 2 ±  0.15 cm^2^/(Vs) and 3.5 ±  0.3 cm^2^/(Vs) are extracted. In Figure S15 (Supporting Information) a plot showing the variation of the most relevant figure‐of‐merits is presented. Figure 5b shows 20 consecutive measurements performed on a short channel OFET, which, differently from before, shows very minor reductions of only ≈1.5% in on‐current and ≈2% in width‐normalized transconductance. In Figure 5c, instead, the results of a bias stress experiment performed at the maximum operating voltages (*V_gs_* = *V_ds_* = ‐10 V) are presented, which led to only a ≈5% reduction in drain current after continuous operation for 2⋅10^3^ s.

**Figure 6:**
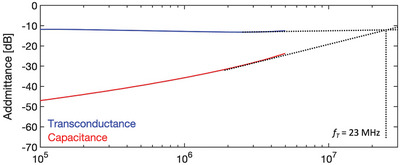
Measurement of channel transconductance and total gate admittance of an OFET characterized by channel and overlap lengths equal to 1.3 and 1.9 µm, respectively, and channel width of 350 µm at *V_gs_* = *V_ds_* = ‐8 V.

After the reduction of the operation voltages and stabilization of OFETs with respect to thermal degradation, channel and overlap lengths were minimized as much as possible to improve the final device dynamic performance. To this purpose, doped transistors with *L_C_* of 1.3 µm and *L_OV_* equal to 1.9 µm were fabricated (e.g., Figure 5e). An example of transfer curve is shown in Figure 5d, while in Figure 5f the corresponding apparent saturation mobility and width‐normalized transconductance are depicted. A value of *µ*_app_ as high as 3 cm^2^/(Vs) is derived, indicating only minor degradation of device performances with respect to transistors with *L_C_* = 2.5 µm. A quasi‐static *g_m_*/*W* of ≈4 mS cm^−1^ is obtained at *V_gs_* = *V_ds_* = −7.5 V. With such value, after calculating a theoretical *C_g_*/*W* of ∼23 pF cm^−1^ considering a parallel plate capacitor model, a predicted transition frequency as high 27 MHz is estimated. To experimentally derive *f_T_* we used the measurement set‐up previously introduced by Perinot et al., which permits to measure separately the channel transconductance and the gate capacitances.^[^ ^56^ ^]^ In Figure S16 (Supporting Information) the measurements of *g_m_*/*W* and *C_g_*/*W* in the frequency domain are shown for a device with *L_C_* = 1.3 µm and *L_OV_* = 1.9 µm at *V_gs_* = *V_ds_* = ‐8 V. Values equal to 3.5 mS cm^−1^ and 25 pF cm^−1^ are derived, which are comparable with the previous estimations. **Figure** **6** instead shows the experimental extraction of *f_T_* as the intersection of the total gate admittance and the channel transconductance, from which a value as high as 23 MHz is obtained, which corresponds to a *f_T_*/V of 2.8 MHz/V. We note that, with a further reduction of the overlap parasitism by patterning the gate electrode, a transition frequency above 200 MHz seems feasible, close to the Ultra High Frequency (UHF) bandwidth (300 MHz – 3 GHz), important for IoT communication purposes.

**Figure 7:**
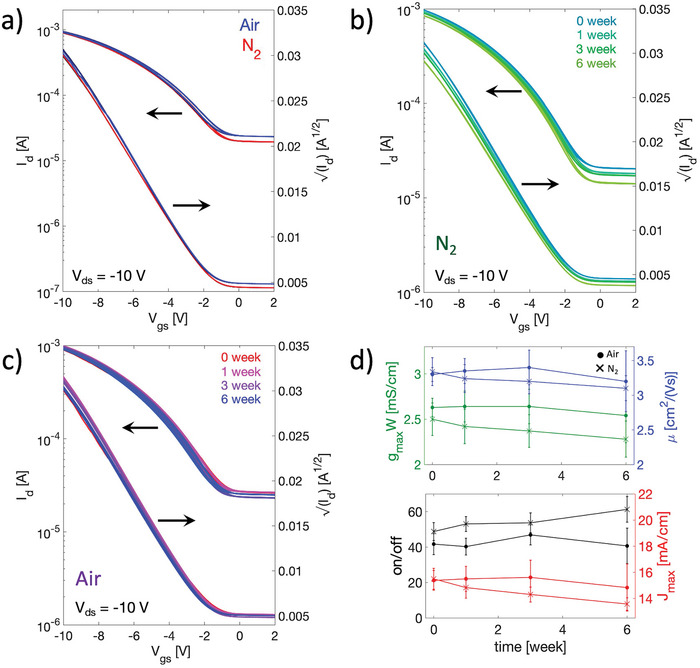
a) Saturation transfer characteristics obtained in air and in glovebox for two short channel doped OFETs (*L_C_* = 2.5 µm) with PVF/Parylene dielectric. Channel width is 600 µm. b) Saturation transfer curves taken in different weeks in a nitrogen atmosphere and c) in air for two representative short channel devices. d) Variation of average values of *J_max_*, on/off ratio, saturation 𝜇_app_ and *g_m_*/*W* with storage time.

Finally, we evaluated the operational stability in air and the shelf‐life of our transistors, which are crucial points for practical applications alongside performances. **Figure** **7**a shows a comparison of the electrical behavior of two representative short channel doped OFETs; one measured in air (blue curve) and the other in a controlled nitrogen atmosphere (red curve). For each device, three consecutive transfer curves were recorded at *V_gs_* = *V_ds_* = ‐10 V. Aside for a small increase of the transistor off current and a slight enhancement of on current, probably due to oxygen doping, no sign of any degradation is visible as the OFETs behave almost identically. This result proves that the transistors have good robustness in air, even without the use of an encapsulation layer. In Figure 7b,c, instead, examples of saturation transfer curves measured during different weeks are reported for two representative OFETs. Figure 7b is relative to a device kept and measured inside a nitrogen glovebox, while Figure 7c to a device stored and measured in air. For each week, three consecutive measurements were performed at *V_gs_* = *V_ds_* = ‐10 V. Figure 7d shows how the most relevant figures of merit (width‐normalized drain current *J_max_*, on/off ratio, apparent saturation mobility and *g_m_*/*W*) change with the storage time, both in air and in nitrogen atmosphere. The extracted parameters are averaged over 10 different transistors. A small decrease in performance is observed when devices are stored inside the glove box, which is likely due to de‐doping phenomena. The on/off ratio is slightly increased, while the maximum on current is reduced with time. Nevertheless, such variation is not particularly severe: −12.5% of *J_max_*, +20.4% of on/off ratio, −7.2% of 𝜇_app_ and −9% of *g_m_*/*W*. However, if the transistors are kept in air, the degradation is delayed, and it occurs after a first improvement in performance, probably due to environmental oxygen doping. In this case after 6 weeks of storage, reduction of only 4% in *J_max_*, 2.4% in on/off, 3% in 𝜇_app_ and 3.5% in *g_m_*/*W* is observed. Therefore, it is possible to conclude that the fabricated OFETs display promising stability already in ambient conditions, which is a very appealing feature for IoT applications, where suitable encapsulation strategies can drastically enhance stability.

## 3 Conclusion and Future Perspectives

In this work the integration of a high‐mobility small molecule/polymer semiconductor blend made of C_8_‐BTBT and C_16_IDT‐BT in downscaled transistor architectures for high‐frequency operation is demonstrated. In particular, we showed the possibility of using such a high‐performing system in OFETs characterized by a 1.3 µm long channel and a 1.9 µm long gate to contacts overlap. The combination of relatively high molecular doping levels (3 mol%) and low voltage operation (≤ 10 V) proved to be two key factors for this result. Doping was fundamental to reduce contact resistance down to 260 Ωcm, allowing to retain a mobility as high as 3 cm^2^/Vs in downscaled transistors. Low voltage operation, made possible with the use of an ultra‐thin organic dielectric stack, was instead crucial to eliminate thermal degradations occurring during the electrical operation. With this strategy, values of transition frequency and voltage‐normalized transition frequency as high as 23 and 2.8 MHz V^−1^, respectively, have been achieved. Such values are among the best results ever obtained with coplanar OFETs, qualifying organic blends as excellent candidates for the development of high‐speed organic electronics, alongside small molecule‐based monolayers. The fabricated devices proved also good stability in ambient conditions, important prerequisite for practical applications. Starting from the reported results and considering further reduction of the gate capacitance by patterning of the gate electrode, back‐of‐the‐envelope estimations indicate the possibility to achieve transition frequencies above 200 MHz at similarly low voltages, getting close to the UHF bandwidth, which would allow the exploitation of OFET based circuits in the IoT field.

## 4 Experimental Section

### Materials and Solution Preparation

C_16_IDT‐BT was synthesized as previously reported, with a M_w_ of 157 Kgmol^‐1^ and Ð of 2.4 as measured by gel permeation chromatography (chlorobenzene, 80 °C) against polystyrene standards. C_8_‐BTBT (≥99% (HPLC), Sigma Aldrich) and C_16_IDT‐BT were both dissolved in chlorobenzene and tetralin (both form Sigma Aldrich) in 1:1 proportion at a concentration of 10 g/L at 80 °C. Then, the C_8_‐BTBT:C_16_IDT‐BT blend solution was prepared by mixing those of the two semiconductors in 1:4 ratio. C_60_F_48_ (provided by Olga Boltalina, Department of Chemistry of the Colorado State University) was dissolved at room temperature in Chlorobenzene at a concentration of 3 g/L by stirring for 3 h. For the doped transistors, a given amount of C_60_F_48_ was added to the blend solution to reach the desired doping level (which was calculated considering the molecular weight of the small molecule and that of the repeating unit of the polymer).

The SAM solution was prepared mixing 7 µL of PFBT (97%, Sigma Aldrich) with 10 mL of isopropyl alcohol (Sigma Aldrich) for a final concentration of 0.0007 *v* *v* ^−1^ at room temperature and stirred for 1 h.

For the dielectric, poly(diallyl dimethylammonium chloride) solution (PDAC, 20 wt.%, Sigma Aldrich) was diluted at 0.5 wt.% in deionized water while poly(vinyl formal) (PVF) (SPI Supplies, trade name Vinylec E Polyvinyl Formal Resin) was dissolved in ethyl lactate (EL, ≥98%, Food Chemical Codex, Food Grade) at concentration of 1 wt.% at 50 °C and stirred overnight. Poly(chloro‐p‐xylene)‐C (Parylene‐C dimer) was purchased from Specialty Coating Systems. PEDOT:PSS (Clevios PJ700 formulation) was purchased from Heraeus.

### OFETs Fabrication

Bottom‐contacts top‐gate transistors were fabricated either on glass substrates (low alkali 1737F Corning glasses, purchased from Apex Optical Services) or on 125 µm thick PEN)substrates (purchased from Du Pont). Source and drain electrodes were defined either by standard photolithography, evaporating 30 nm of gold with a 3 nm thick chromium adhesion layer, or by inkjet‐printed PEDOT:PSS by means of Fujifilm Dimatix DMP2831. Gold contacts were then cleaned by sonication with acetone and isopropyl alcohol (Sigma Aldrich) for 10 and 5 min, respectively, followed by an oxygen plasma treatment (100 W for 5 min). Then, a SAM was formed on gold by immerging the samples for 25 min in the PFBT solution. The blend solution was deposited by spin‐coating in a two‐step procedure (500 rpm for 10 s and 1500 rpm for 30 s). Then the film is dried on a hotplate at 120 °C for 1 min and 45 s. For the low‐voltage OFETs the PVF nanosheet was delaminated in water, according to ref. [55] Parylene was deposited by CVD with a SCS Labcoater 2‐PDS2010 system. Then, a gate electrode in PEDOT:PSS was inkjet‐printed on top at room temperature.

### Static and Dynamic Electrical Characterization

Transfer and output characteristic curves were acquired with a Semiconductor Parameter Analyzer Agilent B1500A for both measurements performed in glovebox (oxygen and water levels below 1 ppm) and in air. The dynamic characterizations were instead carried out using a custom setup which includes an Agilent ENA Vector Network Analyzer and an Agilent B2912A Source Meter. To have more information see reference.^[^ ^56^ ^]^

## Conflict of Interest

The authors declare no conflict of interest.

## Supporting information

Supporting Information

## Data Availability

The data that support the findings of this study are available from the corresponding author upon reasonable request.
