# Supplementary material for: Downscaling of Organic Field‐Effect Transistors based on High‐Mobility Semiconducting Blends for High‐Frequency Operation
Source: Small Methods. 2024 Aug 6;8(12):2400546. doi: 10.1002/smtd.202400546 (PMC11671851; doi:10.1002/smtd.202400546)

Supporting Information

**Downscaling of Organic Field-Effect Transistors based on High-Mobility Semiconducting Blends for High-Frequency Operation**

*Tommaso Losi, Fabrizio Antonio Viola, Elda Sala, Martin Heeney, Qiao He, Hans Kleemann, Mario Caironi**

**Figure S1**

Saturation and linear transfer characteristic curves of long channel OFETs based on pristine C_8_-BTBT:C_16_IDT-BT blend with different contact and substrate types: a) glass substrate with Au/PFBT electrodes, b) glass substrate with PEDOT:PSS electrodes and c) PEN substrate with PEDOT:PSS electrodes.


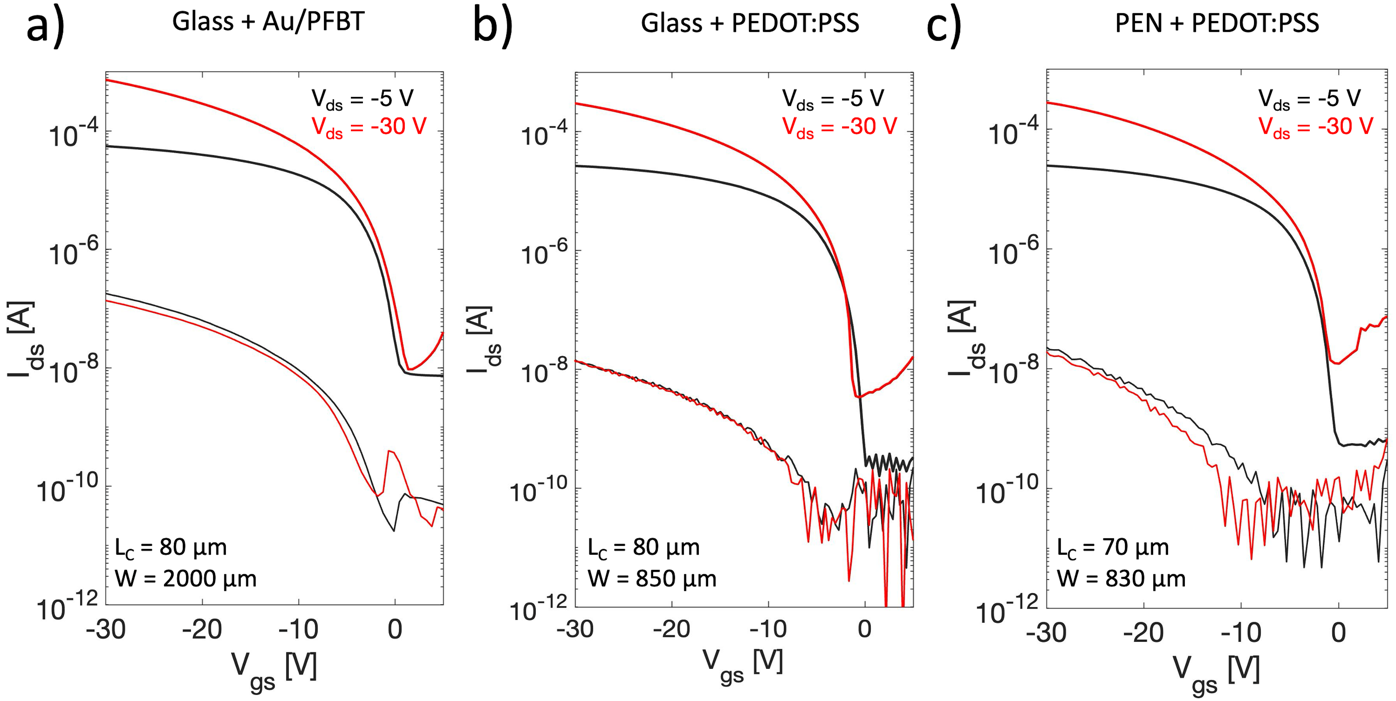


**Figure S2**

Out-put curves of long channel transistors based on pristine C_8_-BTBT:C_16_IDT-BT blend with different contact and substrate types: a) glass substrate with Au/PFBT electrodes, b) glass substrate with PEDOT:PSS electrodes and c) PEN substrate with PEDOT:PSS electrodes.


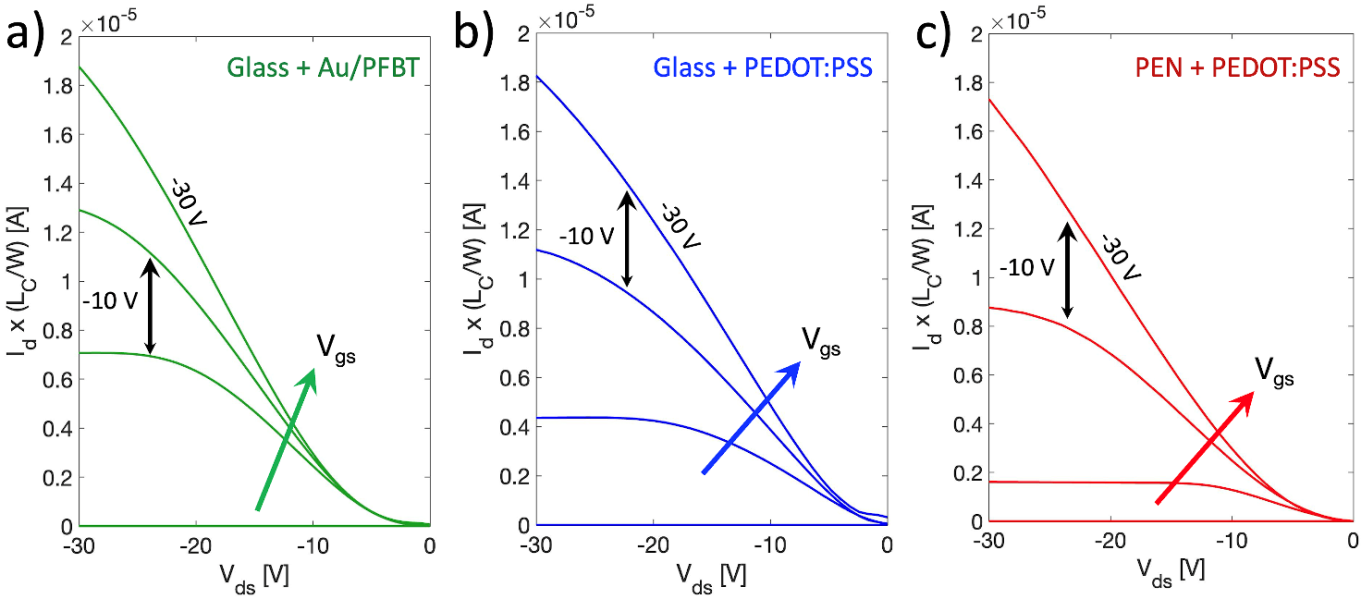


**Figure S3**

a) Saturation and b) linear apparent field-effect mobility curves of long channel transistors based on C_8_-BTBT:C_16_IDT-BT blend with different contact and substrate types (*L_C_* = 80 μm and *W* = 2 mm for glass substrate with Au/PFBT contacts, *L_C_* = 80 μm and *W* = 0.85 mm for glass substrate with PEDOT:PSS contacts and *L_C_* = 70 μm and *W* = 0.83 mm for PEN substrate with PEDOT:PSS contacts).

**
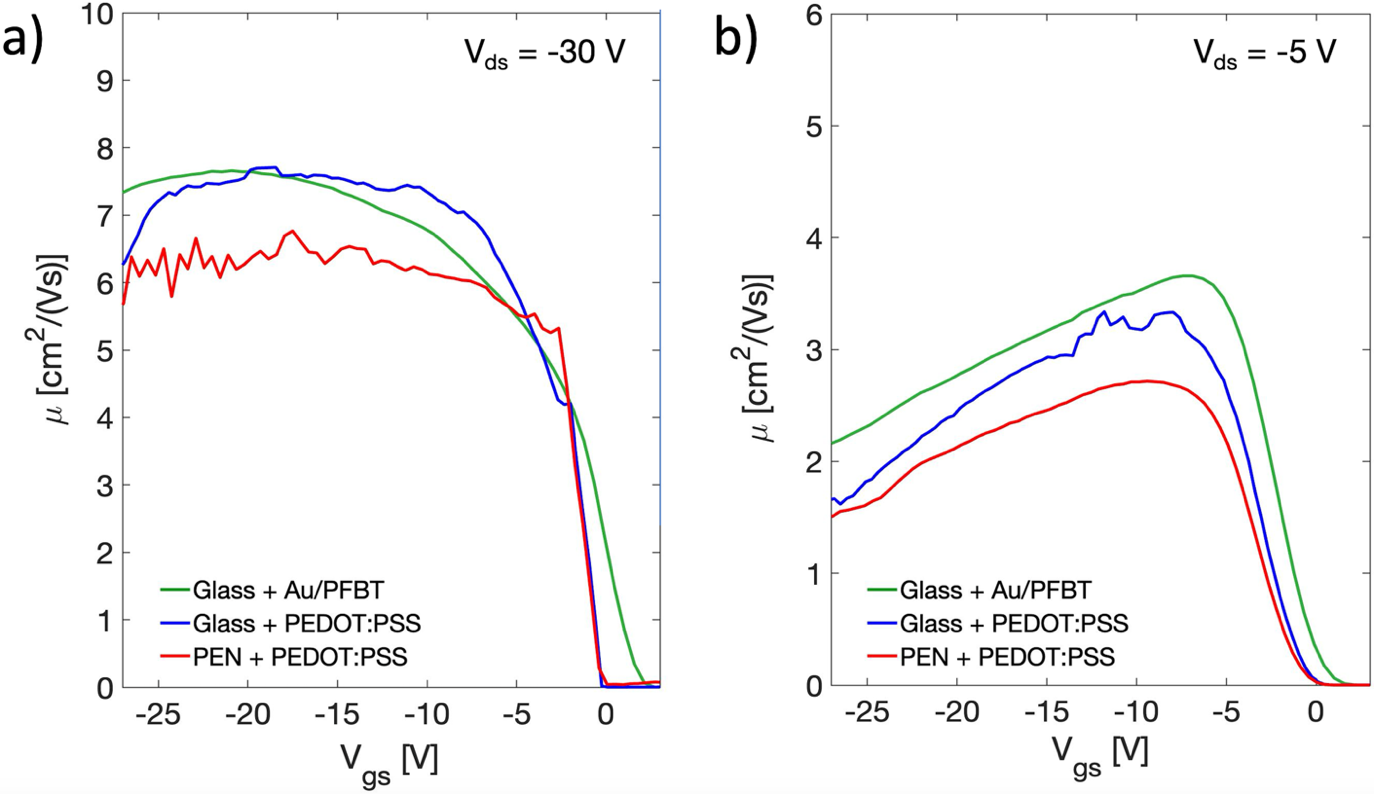
**

**Figure S4**

Direct extraction of contact resistance using the using the Y-function method ^[47]^ from linear transfer curves of long channel transistors having different type of contacts and substrate.

**
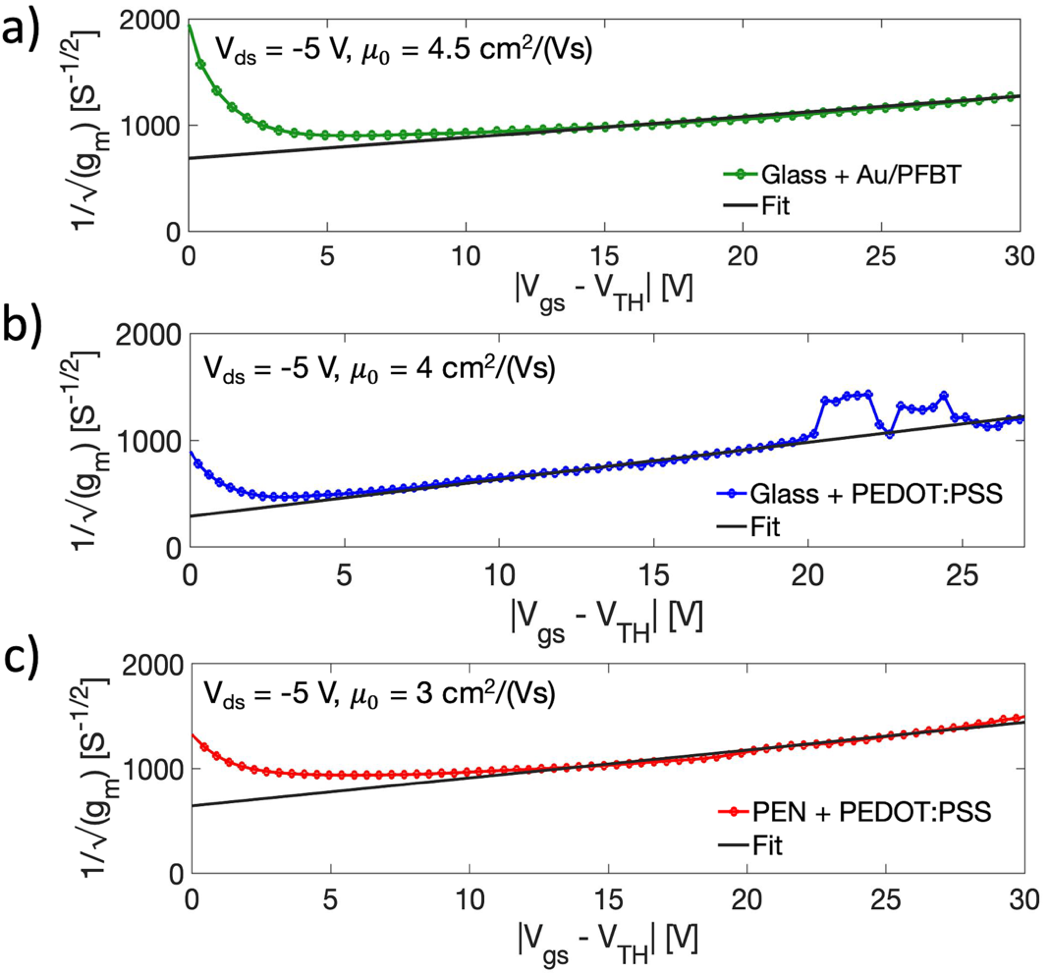
**

**Figure S5**

a) Saturation and b) linear apparent mobility curves of long channel field-effect transistors (*L_C_* = 80 μm and *W* = 2 mm) based on C_8_-BTBT:C_16_IDT-BT with different doping levels (mol% of C_60_F_48_).


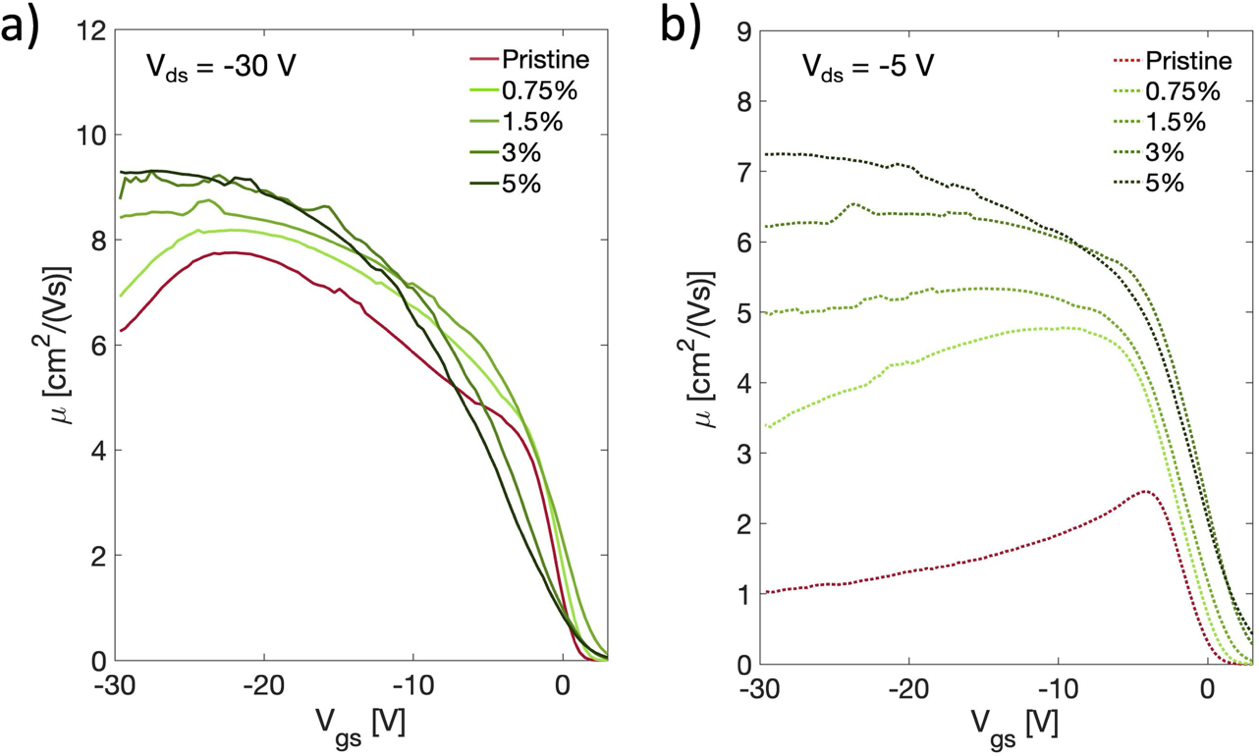


**Figure S6**

Out-put characteristic curves of long channel transistors (*L_C_* = 80 μm and *W* = 2 mm) for different doping levels.

**
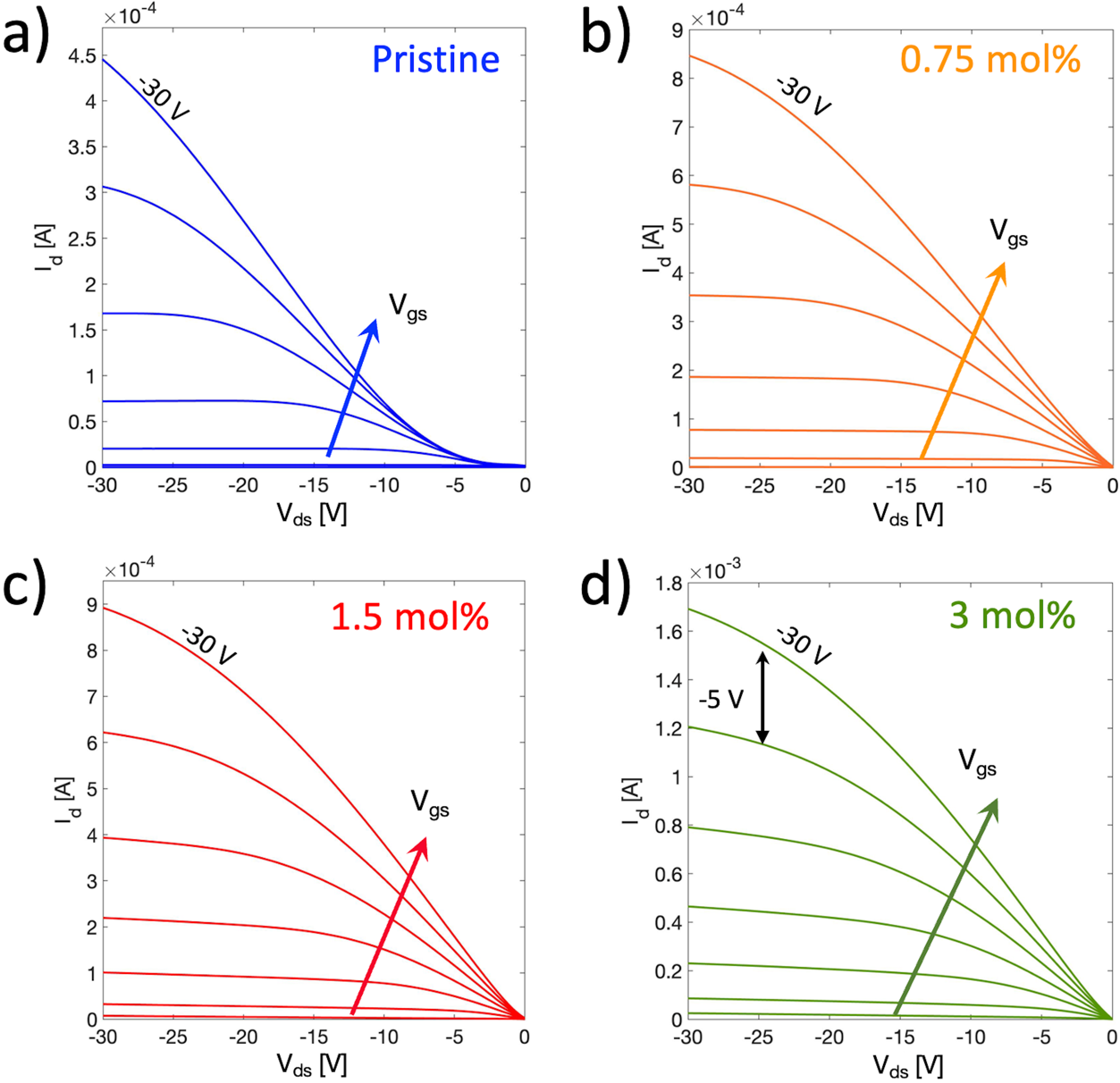
**

**Figure S7**

Example of derivation of contact resistance using the TLM method from a series of OFETs with *W* = 2 mm and different channel lengths (for the doped devices: *L_C_* = 80 μm, 40 μm, 20 μm and 10 μm. For the pristine transistors: *L_C_* = 120 μm, 100 μm and 80 μm). To have a correct extraction of *R_C_W* we verified that drain currents scale properly with *L_C_* in the range of channel lengths considered, and that there is a proper linear dependence with the gate voltage. a) Linear transfer curves of doped transistors having different channel length, b) extraction of contact resistance at different the gate voltages and c) comparison of the injection efficiency in doped OFETs fabricated using an “aged” (storage time in glovebox longer than 3 weeks from preparation) or a “fresh” dopant solution. d) Polarized optical microscope images of different devices changing the channel length.


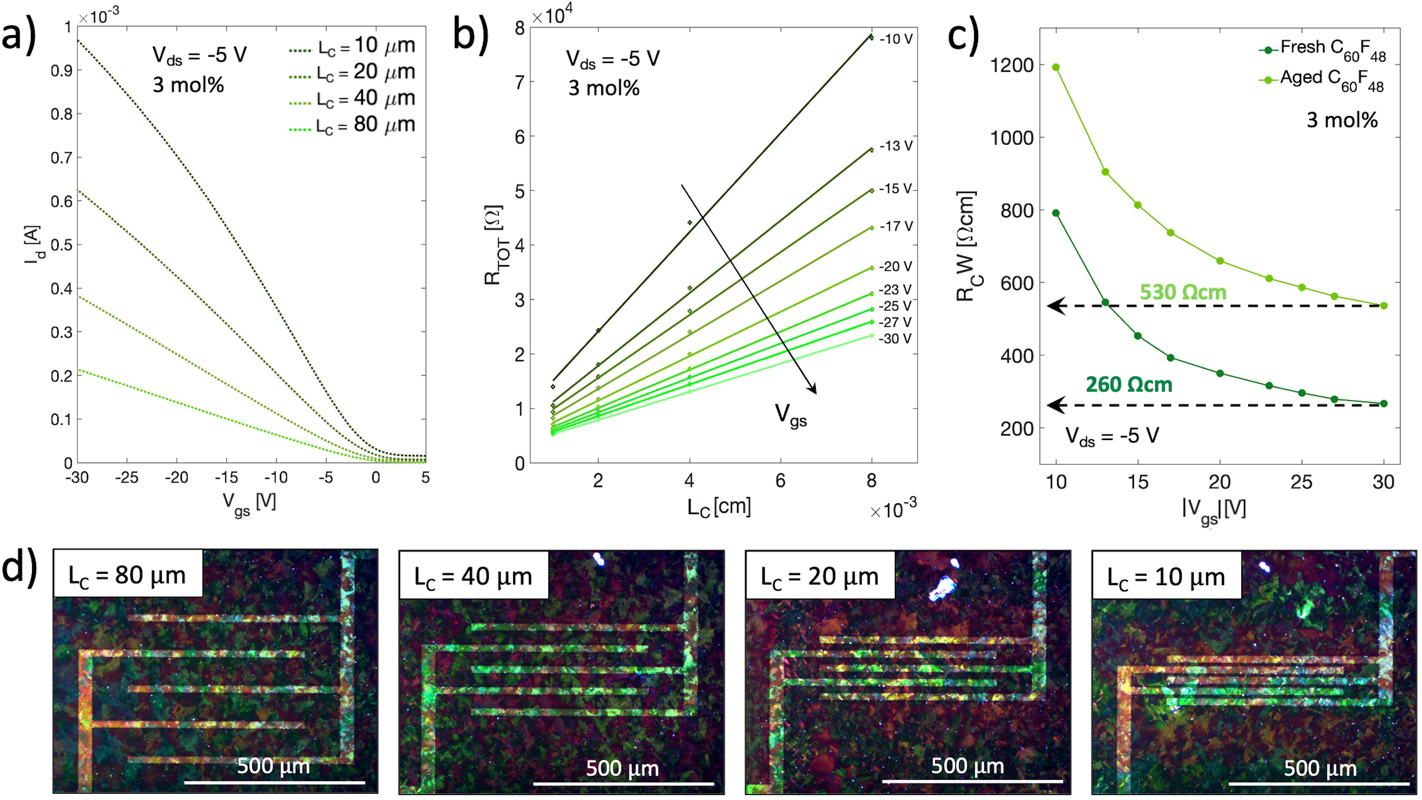


**Figure S8**

Solid-state UV-vis absorption spectra (normalized with respect to the maximum absorption peak) of pristine and doped (10 mol% of C_60_F_48_) thin films of a) C_8_-BTBT and b) C_16_IDT-BT on glass substrates.


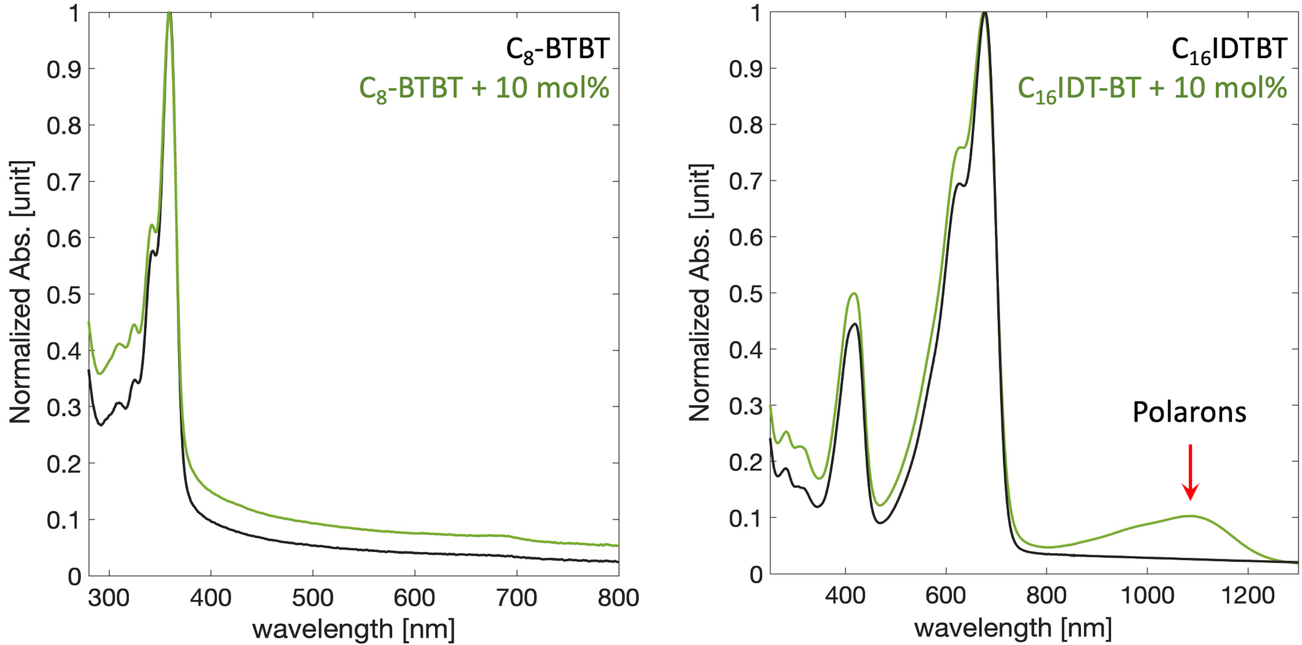


**Figure S9**

Saturation apparent field-effect mobility curves of a) pristine and b) doped (3 mol%) OFETs with *W* = 2 mm and different channel lengths. c) and d) the respective transconductance curves.

**
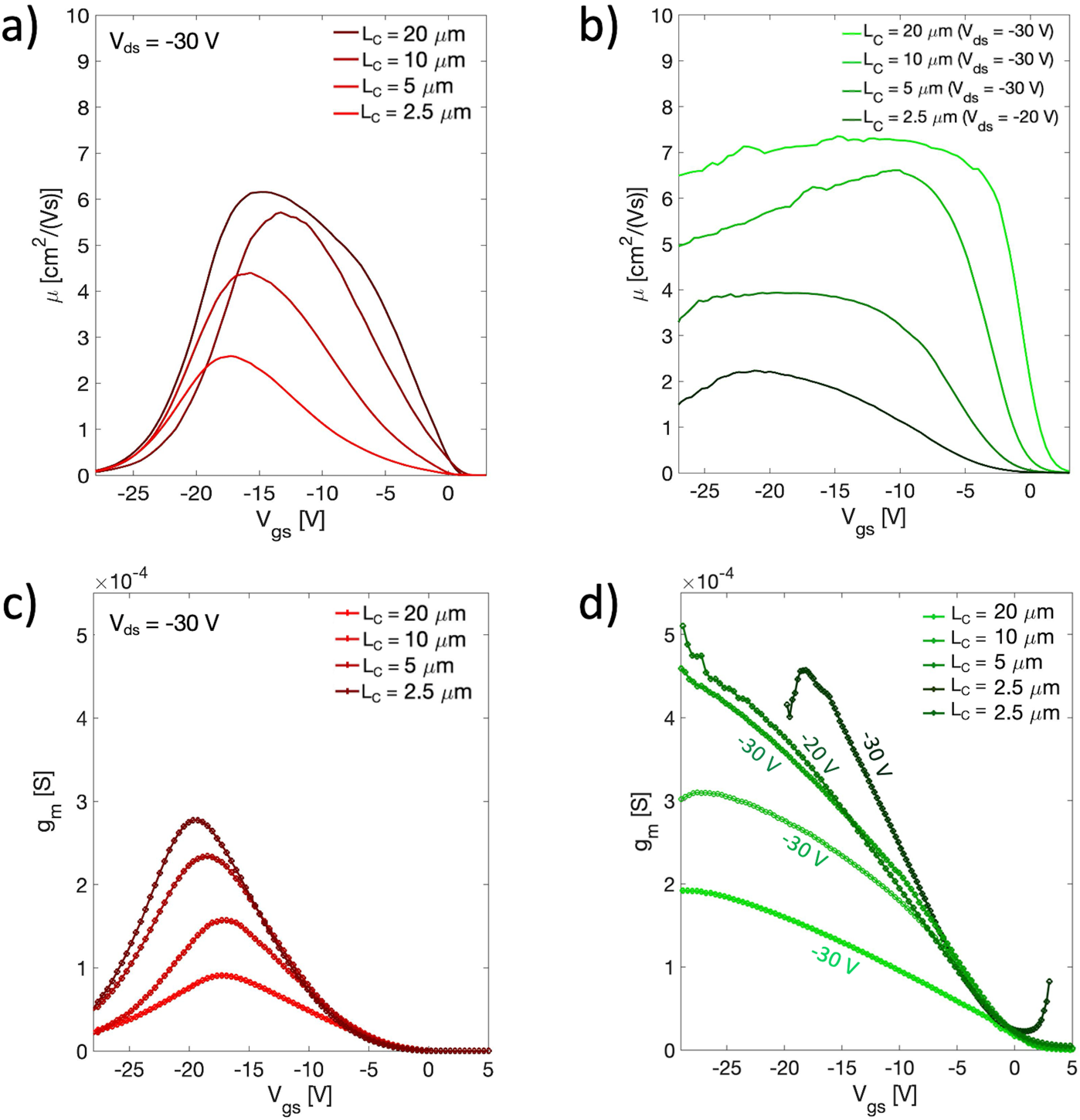
**

**Figure** **S10**

Linear apparent field-effect mobility curves for pristine a) and 3 mol% doped b) transistors having different channel lengths and *W* = 2 mm.


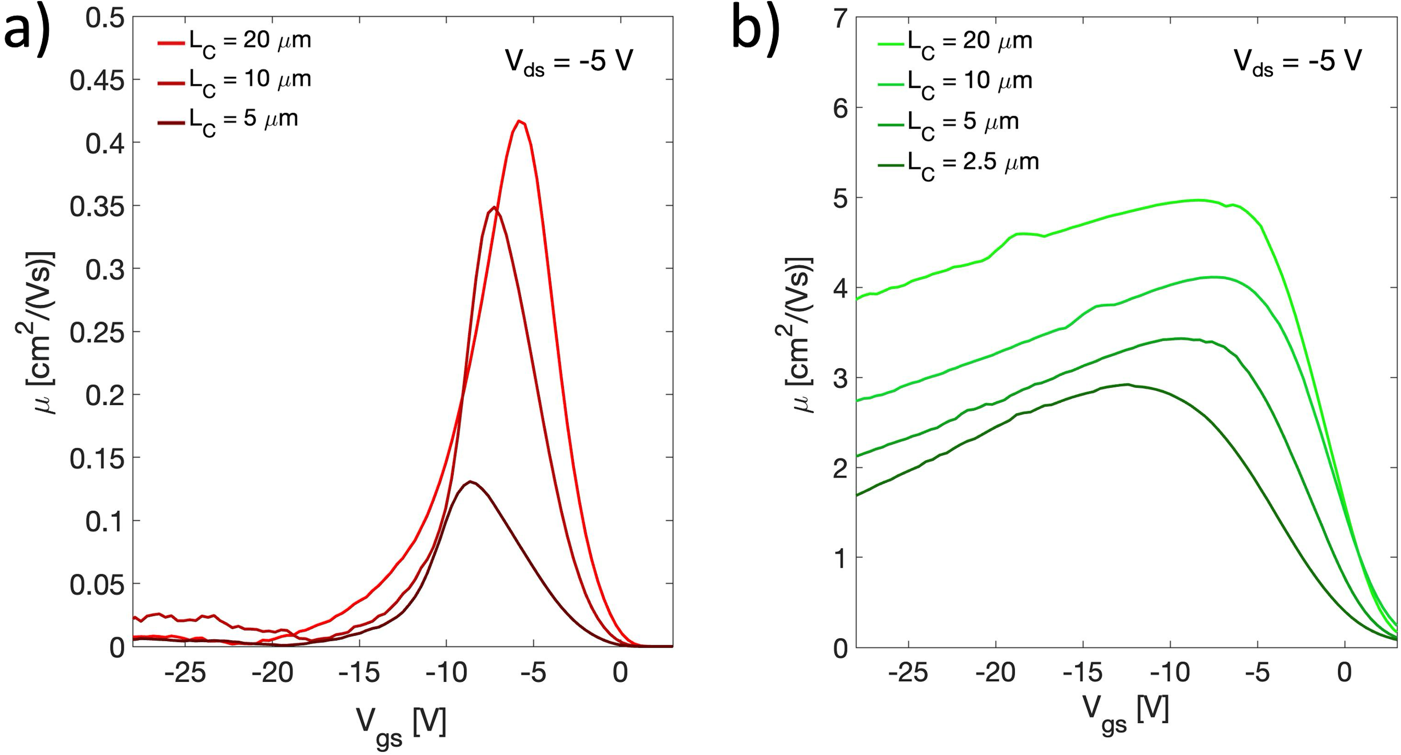


**Figure** **S11**

Transfer length (*L_T_*) was estimated using the following equation: ^[52,53]^

$$R_{rel}=\frac{1}{1+\frac{L_{C}}{2L_{T}}\tanh\left( \frac{L_{OV}}{L_{T}} \right)}$$

where *R_rel_* is the relative contribution of contact resistance with respect to the total device resistance (*R_C_*/[*R_C_* + *R_CH_*]). a) and b) show the graphical solutions of the equation above with F_1_ = 2 ⋅ [(1/*R_rel_*)-1]/(*L_C_*/*L*) and F_2_ = tanh(*L_OV_*/*L*). When F_1_ = F_2_ then, *L* = *L_T_*. For both the pristine and doped blend *L_T_* was extracted using data of transistors for which *R_C_* << *R_CH_*. In particular, the device channel lengths were: 120 μm for the pristine case, while 20 μm for the doped one.

**
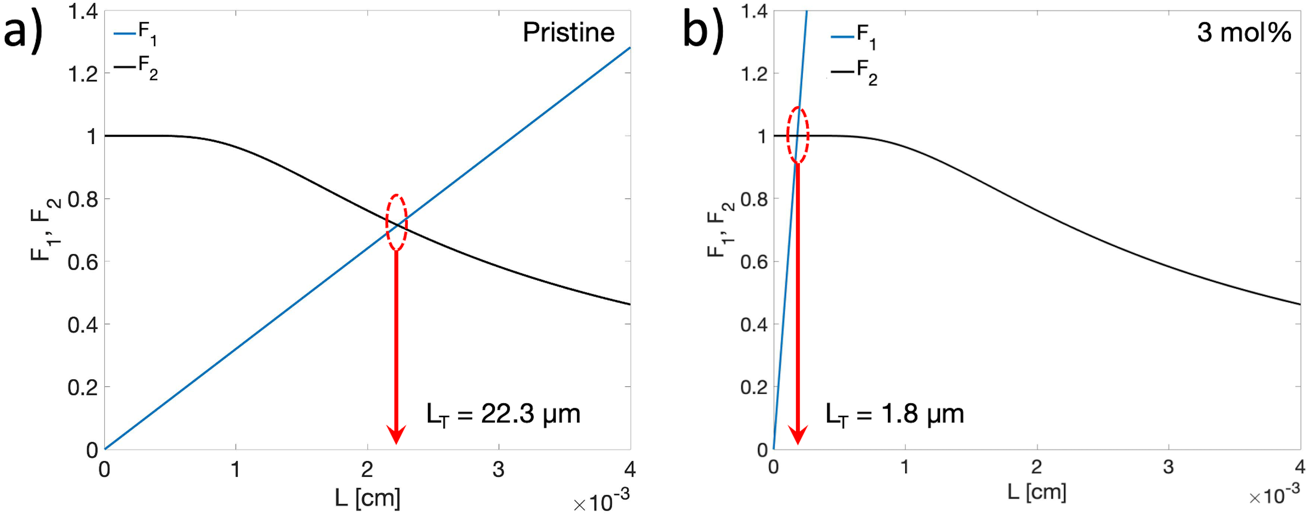
**

**Figure** **S12**

Variation of the most relevant figure-of-merits for ten different downscaled doped (3 mol% of C_60_F_48_) OFETs having a channel length of 2.3 µm and an overlap length of 2 µm. In the figure the modulus of the threshold voltage is reported for both linear and saturation regime.


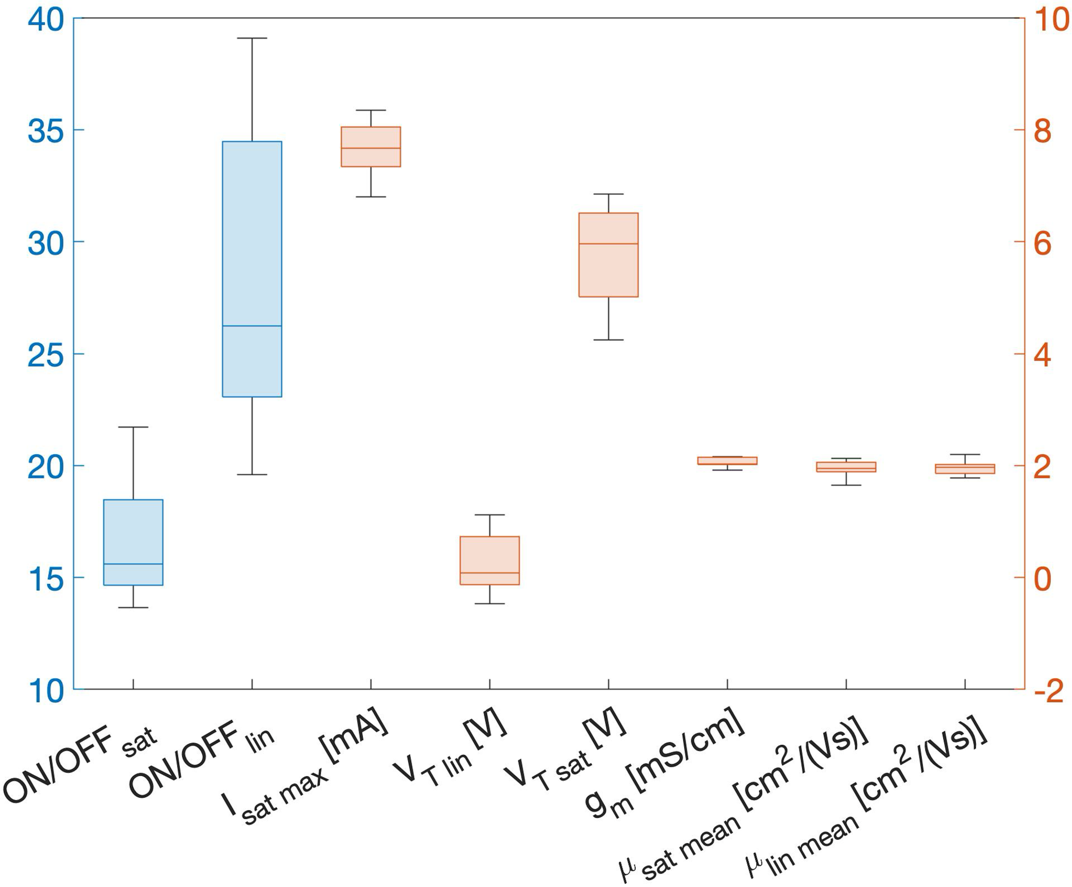


**Figure** **S13**

a) Transfer curves of doped OFETs with *L_C_* of 5 μm (blue curve) and 2.5 μm (green curves), measured at different values of *V_ds_* and in different *V_gs_* intervals. b) Consecutive transfer curves of a 4 μm channel doped OFET at different voltages, acquired every two minutes.


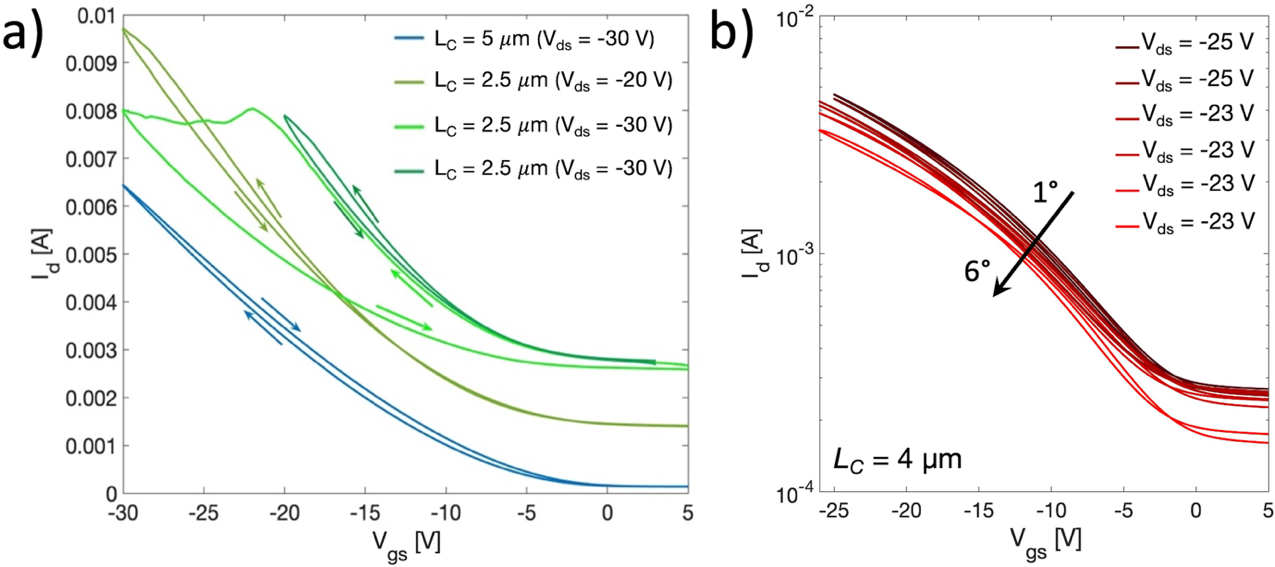


**Figure** **S14**

Saturation transfer curves of doped OFETs (*W* = 2 mm) with a 25 nm PVF nanofilm as dielectric material at different channel lengths measured at a) 5 V and b) 10 V.


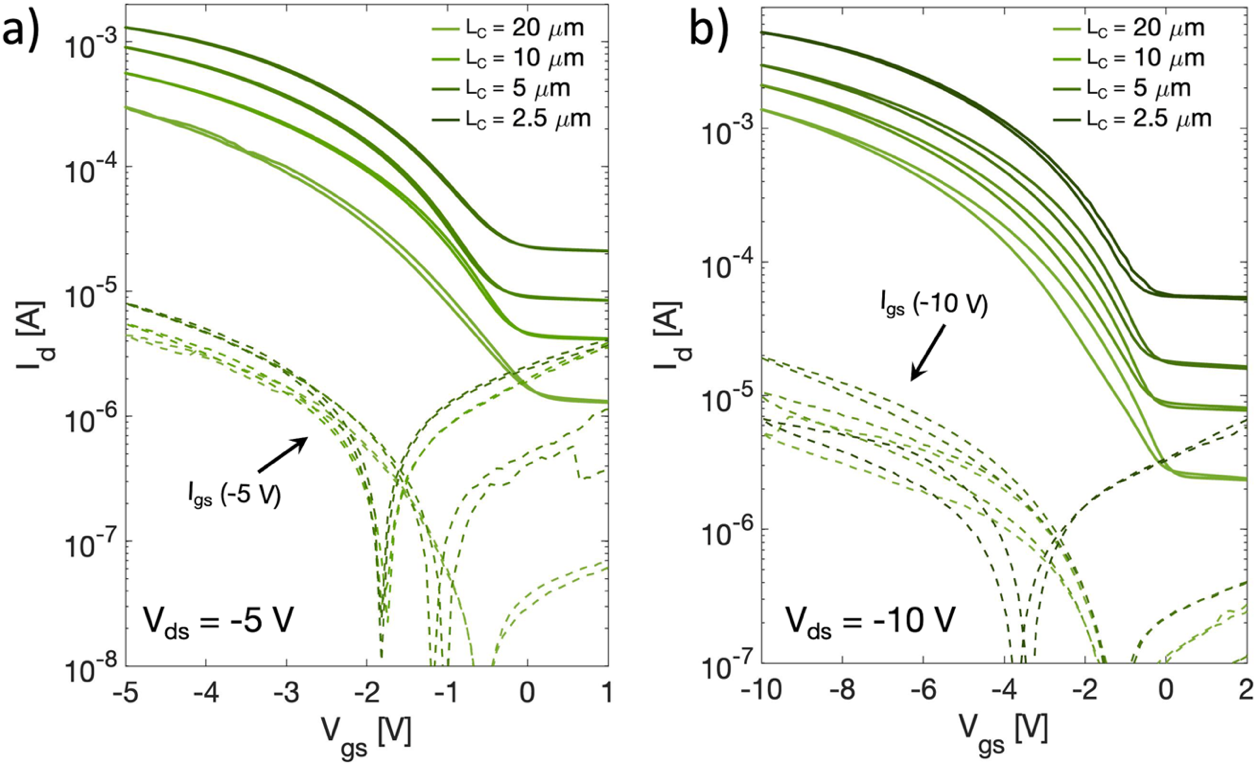


**Figure** **S15**

Variation of the most relevant figure-of-merits for ten short channel doped (3 mol% of C_60_F_48_) transistors (*L_C_* = 2.5 μm) with a double stack dielectric layer. The channel width is 600 µm. In the image the modulus of the threshold voltage is reported for both linear and saturation regime.


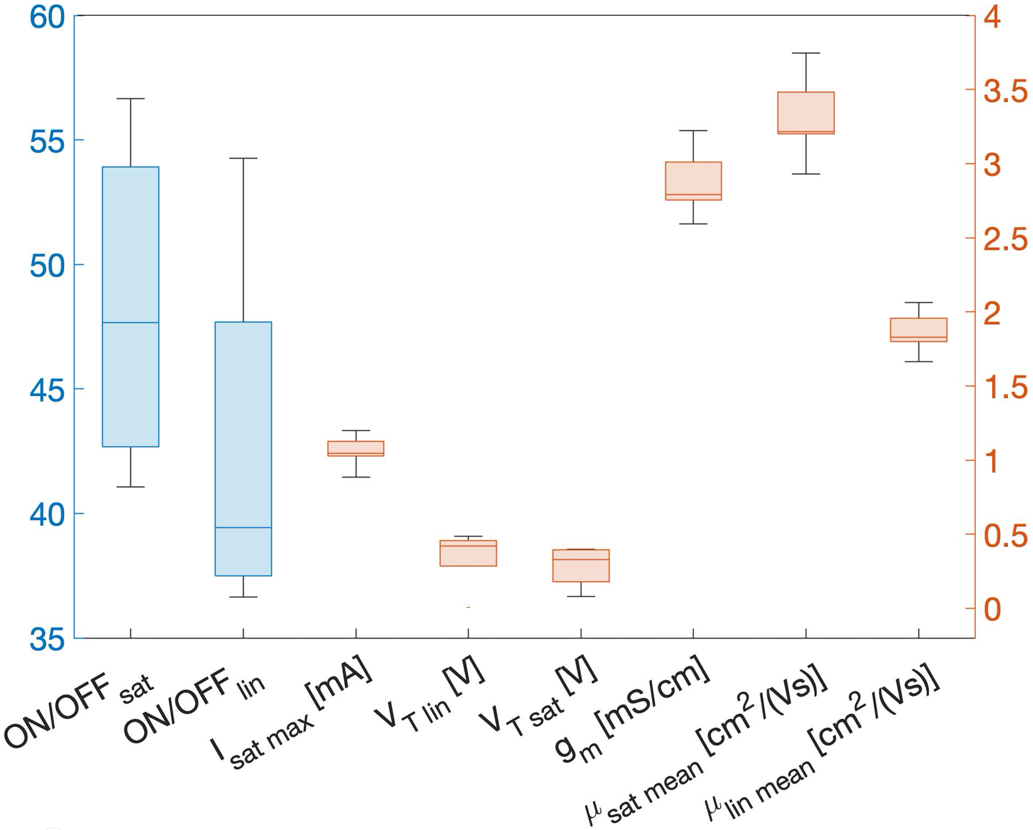


**Figure** **S16**

Dynamic measurements of width-normalized total gate capacitance a) and b) width-normalized transconductance for a doped OFET characterized by *L_C_* = 1.3 µm, *L_OV_* = 1.9 µm and *W* = 350 µm with a PVF/Parylene dielectric.


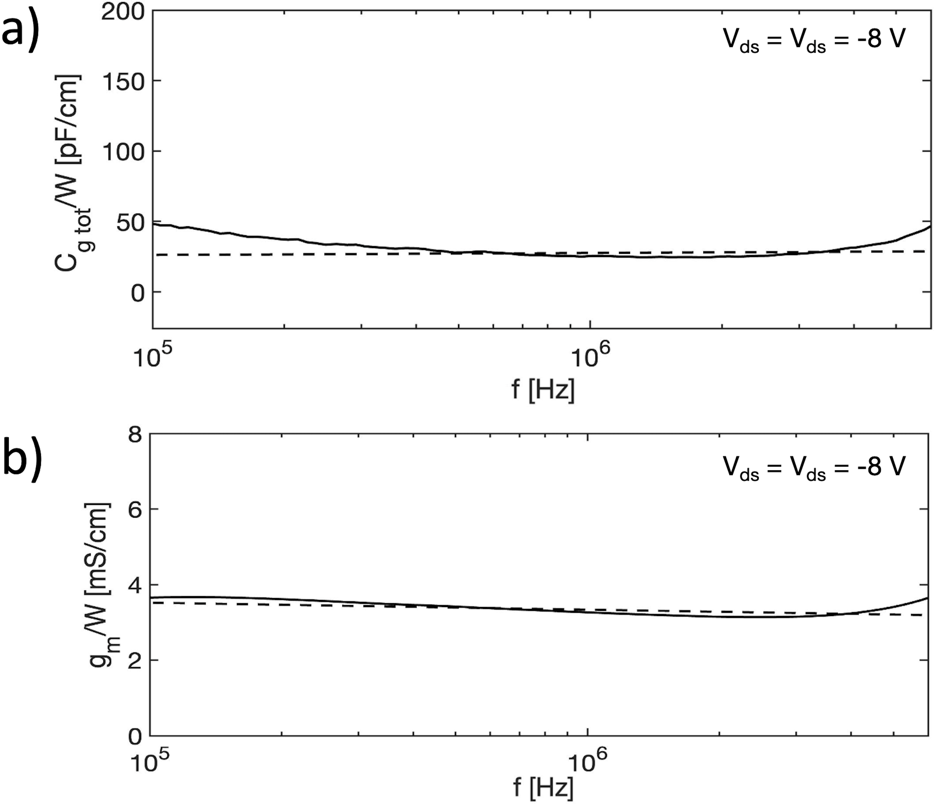

Supplement: Supplementary file 1 — Supporting Information [file SMTD-8-2400546-s001.docx]
